# Impulse Buying and Cognitive Dissonance: Differences in Self-Justification and Symbolic Consumption Among Adult South Korean Generation Z Sports Consumers

**DOI:** 10.3390/bs16060939

**Published:** 2026-06-08

**Authors:** Jiung You, Kwon-Hyuk Jeong

**Affiliations:** 1 Department of Sports Science, School of Creative Arts, Hankyong National University, 327, Jungang-ro, Anseong-si 17579, Gyeonggi-do, Republic of Korea; wldnd6488@hknu.ac.kr; 2 Department of Taekwondo, College of Physical Education, Kyung Hee University, 1732, Deogyeong-daero, Giheung-gu, Yongin-si 17104, Gyeonggi-do, Republic of Korea

**Keywords:** impulse-buying tendency, self-justification, symbolic consumption tendency, Generation Z, sports consumer

## Abstract

This study examined differences in self-justification and symbolic consumption tendency according to levels of impulse-buying tendency among South Korean adult members of Generation Z sports consumers. Drawing on Cognitive Dissonance Theory (CDT) and Symbolic Self-Completion Theory (SSCT), the study aimed to clarify whether impulse-buying tendency functions as a meaningful basis for segmentation in sports product consumption. Data were collected from South Korean adults aged 20 years or older who had purchased sports products within the previous 12 months. For group-based comparison, participants were classified into low (*n* = 128) and high (*n* = 106) impulse-buying tendency groups using a mean-split procedure. A 29-item questionnaire assessed impulse-buying tendency (9 items), self-justification (4 items), and symbolic consumption tendency across five subdimensions (16 items). Confirmatory factor analysis supported the adequacy of the measurement model (*χ*^2^/*df* = 2.285, *p* < 0.001, IFI = 0.918, TLI = 0.906, CFI = 0.918, SRMR = 0.044, RMSEA = 0.074), with satisfactory reliability and convergent validity. MANOVA results showed that the high impulse-buying tendency group reported significantly higher levels of self-justification, self-development and reinforcement, conformity and belonging, and communication and exchange than the low group. These findings suggest that impulse-buying tendency differentiates consumers in terms of post-purchase cognitive responses and identity-related consumption patterns among adult Generation Z sports consumers. The results highlight the heterogeneity of South Korean Gen Z sports consumers and suggest that sports product marketing may benefit from more segmented strategies based on post-purchase reassurance and symbolic value. However, causal interpretations are limited by the cross-sectional design.

## 1. Introduction

Generation Z (hereafter Gen Z) is commonly defined as individuals born between the mid-1990s and the early 2010s and is widely regarded as a cohort of digital natives who have grown up in highly digitalized environments (Chaturvedi et al., 2020). Because the present study included only legal adults aged 20 years or older, however, the empirical focus of the study was limited to the older segment of Generation Z consumers. This generation is deeply embedded in social media, mobile platforms, and real-time interactive environments, and these digitally mediated contexts have structurally reshaped consumption patterns and decision-making processes (Axcell & Ellis, 2023). Compared with previous generations, Gen Z has been reported to respond more sensitively to immediate emotions and environmental stimuli and to exhibit a relatively higher impulse-buying tendency (Lina et al., 2022). Accordingly, understanding the impulsive consumption behavior of adult Gen Z consumers has emerged as an important issue in contemporary consumer research.

Impulse-buying tendency refers to a purchasing tendency characterized by spontaneous buying triggered by emotional stimuli without prior planning, and it is regarded as a relatively stable psychological trait rather than a simple situational response (Rook, 1987). In the context of sports consumption, consumers’ emotional responses are further intensified by the atmosphere of sporting events, fan identity, social interaction, and digital content experiences, all of which increase the likelihood of impulse buying. In this context, impulse buying should not be viewed merely as irrational consumption; rather, it should be understood as a consumption behavior closely connected to sports experiences and identity formation.

Theoretically, impulse-buying tendency has been associated with post-purchase cognitive regulation processes, particularly within Cognitive Dissonance Theory (CDT), which explains how consumers attempt to psychologically justify decisions that generate internal inconsistency (Festinger, 1954). In sports consumption contexts, such post-purchase rationalization may become especially salient because sports products are often closely tied to identity and emotional attachment.

In contemporary sports markets, products increasingly function not only as utilitarian goods but also as symbolic resources for identity expression, social belonging, and self-presentation (Belk, 1988; McCracken, 1986). This dual function is particularly salient among Gen Z consumers who actively construct and communicate identities within digitally mediated environments (Lu et al., 2024). Consequently, impulse-driven sports consumption may extend beyond immediate purchasing behavior and become associated with symbolic identity construction and socially expressive consumption practices.

Although prior studies have identified relationships among impulse buying, cognitive dissonance, and symbolic consumption, most have adopted variable-centered approaches emphasizing linear associations among constructs (Beikverdi et al., 2024; Chen et al., 2023). Consequently, relatively limited attention has been paid to whether consumers with different levels of impulse-buying tendency exhibit qualitatively distinct psychological and symbolic consumption profiles, particularly within sports consumption contexts characterized by strong social visibility and identity expression. In contrast, studies that multidimensionally analyze differences between groups according to levels of impulse-buying tendency remain relatively limited. This gap is particularly evident in sports consumption contexts, where social visibility and identity expression are especially salient.

Accordingly, the present study reconceptualizes impulse-buying tendency as a segmentation criterion. Using MANOVA, this study examines whether impulse-buying tendency is associated with distinct profiles of self-justification and symbolic consumption tendency within South Korean adult Gen Z sports consumers. In addition, South Korean society is characterized by collectivist cultural norms, heightened sensitivity to social evaluation, and highly active interaction in digital environments. This context makes self-expression through consumption and the management of social image particularly salient, thereby strengthening the theoretical and contextual relevance of examining the consumption behavior of Gen Z sports consumers.

## 2. Theoretical Background

### 2.1. Impulse-Buying Theory

Impulse-Buying Theory explains that consumers’ purchasing behavior is not always governed by planned and rational processes, but may also occur spontaneously in response to emotional stimuli and situational cues (Flight et al., 2012). Impulse buying is generally defined as a purchase made in response to an immediate urge without prior planning, and it has traditionally been understood as being closely associated with external stimuli such as store environment, product displays, price cues, and time pressure (Rook, 1987). Subsequent studies, however, have emphasized that impulse buying is not merely a situational response, but is also related to a relatively stable individual disposition, namely impulse-buying tendency (Verplanken & Sato, 2011).

In the context of sports consumption, impulse buying becomes even more salient. Unlike general consumption, sports consumption takes place in an emotionally intensive environment shaped by the combined influence of game attendance, team identification, fan culture, event atmosphere, and digital content consumption (Yim et al., 2021). In particular, Stadium excitement, cheering culture, psychological attachment to teams and players, and real-time online interactions amplify consumers’ emotional responses, prompting spontaneous purchases of merchandise, apparel, and equipment (Wu, 2026). Therefore, impulse buying in sports consumption should be understood not simply as unplanned purchasing, but as a consumption behavior shaped by emotional immersion, social interaction, and fan identity.

In particular, Gen Z is highly accustomed to digital environments, sensitive to real-time information and visual stimuli, and continuously exposed to others’ consumption patterns and lifestyles through social media, all of which may strengthen impulse-buying tendency (Indriastuti et al., 2024). South Korean Gen Z sports consumers are likewise situated in highly connected consumption environments involving online commerce, live commerce, short-form content, and fan community platforms, and these environments facilitate immediate purchasing responses (Jang, 2022). In this regard, impulse-buying tendency may function as a meaningful segmentation criterion within sports consumption contexts. Nevertheless, prior studies have primarily focused on identifying antecedents or outcomes of impulse buying rather than examining whether differing levels of impulse-buying tendency correspond to distinct psychological and symbolic consumption orientations. Collectively, these studies suggest that impulse-buying tendency in sports consumption is shaped not only by immediate emotional reactions but also by socially mediated and identity-related consumption processes.

Because impulsive purchasing frequently involves emotionally driven and less deliberative decision-making—operating predominantly through what dual-process models describe as the impulsive rather than the reflective system (Strack & Deutsch, 2004)—it may also increase the likelihood of post-purchase psychological tension and cognitive regulation processes.

### 2.2. Cognitive Dissonance Theory: Self-Justification in Sports Consumption

Whereas Impulse-Buying Theory characterizes the dispositional and situational antecedents of impulsive purchasing, Cognitive Dissonance Theory clarifies the cognitive processes that follow it. Cognitive Dissonance Theory posits that when inconsistencies arise among an individual’s behaviors, beliefs, attitudes, or self-concept, psychological discomfort is generated, and the individual attempts cognitive adjustment to reduce it (Festinger, 1954). In the context of consumer behavior, cognitive dissonance frequently emerges after purchase, particularly when consumers are uncertain whether their choice was the best one or recognize that alternative options may have been preferable (Sweeney et al., 2000). Such tension may lead to regret, anxiety, and doubts about the legitimacy of one’s decision, and to alleviate this discomfort, consumers tend to reinterpret and justify their decisions (Lee, 2015).

In this context, self-justification functions as the primary mechanism for reducing cognitive dissonance. Because impulse buying characteristically bypasses extensive pre-purchase deliberation, consumers are more likely to experience post-purchase uncertainty about whether their choice was optimal—a condition central to the generation of cognitive dissonance (Festinger, 1954; Sweeney et al., 2000). Self-justification, therefore, refers to the process through which individuals restore psychological balance by positively reconstructing their choices, devaluing unchosen alternatives, or reframing the decision as optimal given the circumstances (Aronson, 1969; Haque et al., 2026). Accordingly, individuals with a higher impulse-buying tendency may engage more intensively in post-purchase self-justification.

In sports consumption contexts, cognitive dissonance-related self-justification may become particularly salient because contemporary sportswear and equipment increasingly function as symbolic markers of lifestyle, exercise commitment, and athlete identity rather than merely as functional products (Loureiro et al., 2024).

For example, consumers who impulsively purchase expensive premium sportswear or professional-grade equipment in a somewhat unplanned manner may experience financial guilt or psychological dissonance due to the unexpected expenditure. However, they may justify their seemingly irrational consumption behavior by actively assigning meaning to the purchase, such as viewing the product as an essential tool for improving athletic performance or as a worthwhile ‘investment’ that demonstrates trend awareness and expertise within the sports community to which they belong (Funk et al., 2022). In other words, in participatory sports consumption, self-justification extends beyond merely reducing post-purchase cognitive discomfort and becomes a key mechanism for maintaining a high level of emotional commitment to exercise and defending the social identity of being a ‘professional and passionate sport participant’ that the individual aspires to embody.

Taken together, previous studies consistently indicate that impulsive purchasing is associated with heightened post-purchase cognitive tension and rationalization processes. However, relatively limited research has examined whether the intensity of self-justification differs systematically across consumers with different dispositional levels of impulse-buying tendency. Importantly, self-justification as theorized by CDT operates primarily at the cognitive level—restoring internal consistency through post hoc reinterpretation of the purchase decision. This cognitive regulation process, however, does not fully account for the behavioral and expressive responses that may follow impulsive consumption. Specifically, once consumers have cognitively stabilized a purchase decision, they may subsequently seek to affirm the social and identity-related meaning of that decision through symbolic consumption practices. This suggests that CDT and Symbolic Self-Completion Theory (SSCT), rather than functioning as independent explanatory frameworks, may address complementary but distinct psychological layers of the same impulsive consumption episode—the former governing cognitive consistency restoration, and the latter governing identity-level compensation and expression.

### 2.3. Symbolic Self-Completion Theory in Sports Consumption

Whereas CDT addresses the cognitive dimension of post-purchase regulation, SSCT (Gollwitzer et al., 1982; Wicklund & Gollwitzer, 1982) addresses a conceptually distinct but complementary dimension: the identity-level response to perceived self-concept gaps. Specifically, SSCT proposes that when individuals perceive a discrepancy between their current self and a desired identity, they tend to rely on symbolically meaningful behaviors or objects to compensate for that incompleteness (Mandel et al., 2017). In the context of impulsive consumption, this identity-compensatory process may operate in parallel with cognitive dissonance reduction: while self-justification restores internal cognitive consistency, symbolic consumption restores external identity coherence by communicating a desired self-image through socially visible products (Carr & Vignoles, 2011). Accordingly, the two theories do not merely co-apply to impulse buying; rather, they address sequentially layered psychological responses—cognitive stabilization followed by identity expression—that high impulse-buying tendency consumers may be more likely to exhibit.

The sports consumption environment is one of the domains in which SSCT is expressed most dynamically. Building on the symbolic function of sports products established above, SSCT provides a mechanism through which consumers actively use these resources to bridge gaps between actual and desired identities (Gollwitzer et al., 1982; Wicklund & Gollwitzer, 1982).

For example, preferring premium sportswear with high entry barriers or insisting on advanced professional equipment that has not been widely popularized cannot be understood solely as behavior intended to improve athletic performance (Y. Kim et al., 2022). Rather, such behavior constitutes a form of symbolic practice through which consumers attempt to demonstrate to others that they hold a superior position within a sports community or possess the identity of an authentic athlete. Accordingly, sportswear consumption should be understood as more than the acquisition of material goods; it serves as a key psychological defense mechanism through which individuals compensate for a deficient self and socially complete the distinctive identity they aspire to attain (Verplanken & Sato, 2011).

In this context, Gen Z is a group for whom the process of symbolic self-completion may be especially pronounced. Gen Z tends to place high value on self-expression, individuality, social recognition, and visibility in digital spaces, and its members are accustomed to constructing and communicating their images through consumption (Shin et al., 2021). Among South Korean Gen Z sports consumers, sports consumption may carry even stronger symbolic meaning within peer groups, online communities, and social media-based comparison cultures (Yim et al., 2021). In this sense, the purchase and use of sports products are not merely behaviors aimed at obtaining functional satisfaction, but may also serve as a means of signaling who one is and to which group one belongs (Guo et al., 2023).

The present study draws on SSCT to interpret why groups with higher levels of impulse-buying tendency may differ in symbolic consumption tendency. Impulsive consumption does not necessarily remain limited to unplanned or irrational purchasing; rather, it may subsequently function to make consumers appear closer to a desired identity or to connect them with a socially meaningful image. In contexts such as sports consumption, where visibility is high and symbolic meaning is strong, consumers may be especially likely to use products to compensate for perceived deficiencies in self-concept and to complete their identities. Therefore, this theory provides a meaningful theoretical framework for explaining group differences in symbolic consumption tendency.

Recent studies further confirm that symbolic consumption among younger consumers increasingly operates through digitally visible identity signaling and platform-based self-presentation (Lu et al., 2024), reinforcing the relevance of SSCT in digitally mediated sports consumption contexts. Taken together, the present study conceptualizes CDT and SSCT as addressing two distinct but theoretically interconnected psychological pathways activated by high impulse-buying tendency: (1) a cognitive regulation pathway, in which post-purchase dissonance motivates self-justification to restore internal consistency; and (2) an identity compensation pathway, in which perceived self-concept gaps motivate symbolic consumption to reinforce and communicate desired identities. Both pathways are theorized to be more pronounced among consumers with higher impulse-buying tendency, as their characteristically less deliberative purchasing decisions generate greater cognitive and identity-level tension requiring resolution. Although the cross-sectional design of the present study does not permit causal or temporal ordering of these pathways, their conceptual distinction provides a theoretically grounded basis for examining group differences in self-justification and symbolic consumption tendency as separable but related outcomes.

## 3. Research Hypothesis

### 3.1. Differences in Self-Justification According to Impulse-Buying Tendency

Impulse buying is understood not merely as unplanned purchasing behavior, but as a consumption tendency shaped by the combined influence of emotional gratification and the attribution of social meaning (Dittmar et al., 1995). In particular, consumers with a higher impulse-buying tendency are more likely to be sensitive to immediate emotional responses and to exhibit relatively lower deliberative control (Duan, 2025). These characteristics may lead to a stronger cognitive tendency to justify one’s choices after purchase (Akbar et al., 2020).

As theorized in Section 2.2, the limited deliberation inherent in impulse buying elevates post-purchase dissonance, which consumers regulate through self-justification (Akbar et al., 2020; Saranya & Joji Alex, 2024). To reduce such discomfort, consumers may engage in self-justification by positively reinterpreting their choices or perceiving the purchase as the best possible decision under the circumstances (Saranya & Joji Alex, 2024). This tendency may be particularly pronounced in sports consumption, where product purchases are closely tied to identity, fan experience, and emotional involvement rather than being merely economic choices. Given the collectivist and digitally hyperconnected context of South Korean Gen Z consumers described in the Introduction, social visibility further amplifies this post-purchase justification tendency.

Accordingly, consumers with a higher impulse-buying tendency are expected to exhibit higher levels of self-justification than those with a lower impulse-buying tendency. Therefore, the following hypothesis is proposed.

**H1.**  *Gen Z sports consumers with a higher impulse-buying tendency will exhibit higher levels of self-justification than those with a lower impulse-buying tendency.*

H1 addresses the cognitive regulatory response to impulsive consumption, as theorized by CDT. Building on the dual-pathway framework outlined in Section 2, H2 addresses a conceptually distinct but related identity-level response. While self-justification (H1) restores internal cognitive consistency following an impulsive purchase, symbolic consumption (H2) is theorized to restore external identity coherence by using products as visible symbolic resources. Both hypotheses share a common dispositional antecedent—impulse-buying tendency—but are grounded in theoretically separable psychological mechanisms.

### 3.2. Differences in Symbolic Consumption Tendency According to Impulse-Buying Tendency

Next, from the perspectives of symbolic self-completion and symbolic consumption, consumers with a higher impulse-buying tendency are more likely to respond sensitively to external stimuli and social signals and to use consumption not merely for functional acquisition but also as a means of self-expression and identity communication (Dittmar et al., 1995). In the context of sports consumption, products carry symbolic meanings related to team belonging, fan identity, individuality, trendiness, and social image. Accordingly, groups with a higher impulse-buying tendency may also exhibit higher levels of symbolic consumption tendency (Rincón et al., 2023).

This tendency is expected to be especially pronounced within the South Korean Gen Z context for reasons outlined in Section 1. Through sports-related products, they may seek to express themselves, signal belonging to specific groups, and construct socially meaningful images. In addition, impulsive purchasing in sports consumption contexts may be psychologically linked to identity-related motives. Because sports products frequently function as visible symbolic signals associated with lifestyle, belonging, and self-image, consumers with stronger impulse-buying tendencies may rely more heavily on symbolic consumption practices to reinforce or communicate desired identities.

Therefore, consumers with a higher impulse-buying tendency may show higher levels across the subdimensions of symbolic consumption tendency, including self-development and reinforcement, conformity and belonging, self-formation, communication and exchange, and judgment and evaluation.

Based on these theoretical and empirical considerations, this study expects significant differences in symbolic consumption tendency and its subdimensions according to levels of impulse-buying tendency. Accordingly, the following hypotheses are proposed.

**H2.**  *Gen Z sports consumers with a higher impulse-buying tendency will exhibit higher levels of symbolic consumption tendency than those with a lower impulse-buying tendency.*

## 4. Materials and Methods

### 4.1. Study Design

To test H1 and H2, this study examined whether South Korean Generation Z sports consumers differ in self-justification and symbolic consumption according to their impulse-buying tendency. The investigation adopted a quantitative framework utilizing a convenience sampling strategy. Eligible respondents were administered a web-based survey that comprehensively outlined the study’s purpose, procedural guidelines, and ethical considerations. The empirical data were gathered over an approximate two-month timeframe, spanning from 16 June to 25 August 2025.

### 4.2. Participants

The survey participants were adults from South Korea who were at least 20 years old and had purchased sports products within the past year. This included Gen Z members (those born between 1995 and early 2010s). Participants were recruited from five universities, seven sports centers, and two golf facilities in South Korea. Because recruitment was conducted exclusively through five universities, seven sports centers, and two golf facilities, the final sample is best characterized as a purposive convenience sample of physically active, sport-engaged Gen Z consumers, rather than a probability sample of the broader Gen Z population. Three structural features of this recruitment design merit explicit consideration when interpreting the findings. First, the sample is likely to overrepresent consumers with pre-existing interest in sports, who may attribute greater symbolic and identity-relevant meaning to sports products than non-enthusiast Gen Z consumers. Second, university-based recruitment may have disproportionately captured Gen Z consumers with comparable educational attainment, peer-network exposure, and discretionary spending profiles, while underrepresenting non-student Gen Z consumers (e.g., early-career workers, vocational trainees, or socioeconomically disadvantaged subgroups) whose consumption motives may differ. Third, recruitment through sport facilities likely captured consumers who have already committed to a sport as an identity-relevant practice, potentially amplifying symbolic consumption tendencies relative to casual or non-participatory Gen Z consumers. Accordingly, the symbolic consumption patterns reported here should be interpreted as estimates within an identity-engaged subpopulation, which may be more pronounced than those observable in broader Gen Z populations including sedentary or exclusively online sports media consumers. The primary analytic focus was on intra-generational heterogeneity (high vs. low impulse-buying tendency) within Gen Z, as a relatively homogeneous age cohort helps isolate differences associated with impulse-buying tendency from age or cohort related effects. Accordingly, the findings should be interpreted as evidence of within-cohort variation in consumer psychology and sports product consumption patterns among South Korean adult Gen Z consumers, rather than as estimates intended for broad population generalization. Therefore, the findings should not be interpreted as representative of the entire Generation Z population, particularly adolescents and financially dependent minors whose purchasing autonomy and consumption opportunities may differ substantially from those of adult consumers.

A total of 250 individuals initially participated in this study. Because recruitment was conducted primarily through universities and physical activity venues, the sample largely comprised physically active Gen Z sports consumers. After excluding 16 incomplete or unreliable responses, 234 valid cases were retained for the final analyses.

This study was approved by an IRB. To conduct the survey, an online questionnaire was used. This included information on the study and participation. Before starting the survey, participants were presented with an online information sheet and were required to provide electronic informed consent by checking an “I agree to participate” box to proceed. The questionnaire was distributed through university club presidents, student association leaders, and staff at sports centers and sports facilities via QR codes and web links. Participation was entirely voluntary, and no incentive or pressure was applied. All the information that the people participated in was kept confidential. The survey was completed anonymously (no personally identifying information was collected), and participants were reminded that there were no right or wrong answers to reduce evaluation apprehension and socially desirable responding. Non-participation carried no academic, social, or institutional consequences for prospective respondents.

### 4.3. Data Collection Tools

To identify the demographic characteristics of the respondents, four background variables were assessed: gender, prior impulse-purchase experience related to sports products, primary retail channel, and main type of sport participation. In addition, the principal psychological variables relevant to sports product consumption were measured, including impulse-buying tendency, self-justification, and symbolic consumption tendency.

Impulse-buying tendency was used as the independent variable for comparative analysis. The impulse-buying tendency factor was measured as a single-factor construct comprising nine items, based on the scale developed by Verplanken and Herabadi (2001). In this study, impulse-buying tendency was operationalized as a relatively stable consumer disposition toward unplanned and spontaneous purchasing driven by emotional triggers rather than cognitive deliberation. This operational choice was made to support clear group-based comparisons aligned with the study aim, namely, the examination of intra-generational differences within South Korean Gen Z sports consumers. The original scale was adapted to fit the context of sports product consumption while preserving its conceptual integrity.

This study also used a modified self-justification scale to assess consumers’ post-purchase cognitive responses. Specifically, self-justification was measured as a single-factor construct comprising four items adapted from prior studies on post-decision dissonance and self-justification (Knox & Inkster, 1968; Sweeney et al., 2000). The scale was designed to capture the extent to which consumers justified the sports product they selected, perceived their decision as the best option at the time, devalued alternative products, and focused on the positive attributes of the purchased product. Minor wording modifications were introduced to ensure suitability for the context of sports product consumption.

Subsequently, symbolic consumption tendency was measured using an adapted multidimensional scale based on prior research in symbolic consumption and consumer behavior (Jeong & Jeon, 2020; J. Kim, 2022; McCracken, 1986; Piacentini & Mailer, 2004). The scale comprised five constructs: self-development and reinforcement (SR; five items), conformity and belonging (CB; three items), self-formation (SF; three items), communication and exchange (CE; three items), and judgment and evaluation (JE; two items). In this study, symbolic consumption tendency was operationalized as consumers’ tendency to use sports product consumption as a means of expressing identity, values, social image, and group belonging. The instrument was adapted and refined to reflect the sports product consumption context while maintaining the conceptual structure of the original measurement model.

All survey items employed a seven-point Likert scale ranging from one point (“Not at all”) to seven points (“Very much so”).

### 4.4. Data Analysis

The empirical data, comprising 234 valid responses, were analyzed utilizing SPSS version 27.0 and Amos version 22.0 The analytical procedure was executed in several sequential phases. Demographic profiles were summarized using frequency distributions. Construct validity and internal consistency of the measurement model were assessed through confirmatory factor analysis (CFA) and Cronbach’s α. Means, standard deviations, skewness, and kurtosis were computed for all constructs, and a Pearson correlation matrix was generated to examine bivariate associations.

Before running MANOVA, key statistical assumptions were tested. Univariate normality was assessed using skewness and kurtosis indices of the dependent variables, and multicollinearity was examined through their intercorrelations. Furthermore, the homoscedasticity of covariance matrices was validated via Box’s M test, and Levene’s test was applied to ensure the equivalence of error variances across the specified cohorts.

Ultimately, the sample was bifurcated into high and low impulse-buying groups based on their respective propensity scores. To ascertain whether self-justification and the five distinct subdimensions of symbolic consumption varied significantly between these two segments, a one-way multivariate analysis of variance (MANOVA) was administered. Upon detecting a statistically significant multivariate omnibus effect, post hoc univariate ANOVAs were deployed to pinpoint the specific dependent constructs driving these group disparities. An alpha level of *p* < 0.05 was established as the criterion for statistical significance across all analyses.

## 5. Results

### 5.1. Participants’ Basic Characteristics

The required sample size for this study was determined using G*Power version 3.1.9.7 prior to data collection. Because the study aimed to examine group differences across six dependent variables, a power analysis for MANOVA global effects with two groups and six dependent variables was conducted. Assuming a medium multivariate effect size, *f*^2^(*V*) = 0.0625, an alpha level of 0.05, and statistical power of 0.80, the minimum required sample size was estimated to be 226. A total of 250 questionnaires were initially collected, and after excluding 16 incomplete or unreliable responses, 234 valid cases were retained for the final analyses.

To enable comparative evaluations via MANOVA, the aggregate sample was dichotomized into high and low impulse-buying cohorts utilizing a mean-split criterion based on the overall average (M = 3.40). Participants scoring above this threshold formed the high impulse-buying group (*n* = 106), while those scoring below formed the low impulse-buying group (*n* = 128). This grouping enabled theoretically grounded comparison of self-justification and symbolic consumption across impulsivity levels. Such an approach strongly aligns with established methodological traditions of market segmentation within consumer behavior literature (Dhandra, 2020; Rook & Fisher, 1995).

The purpose of the present study was not to estimate the linear predictive magnitude of impulse-buying tendency on consumer outcomes, but rather to examine whether distinct psychological and symbolic consumption profiles emerge across consumers with different levels of impulse-buying tendency. Accordingly, a segmentation-based MANOVA framework was considered conceptually appropriate because it allows the identification of dispositional heterogeneity and comparative group patterns within a single generational cohort. In this respect, the analytical focus of the present study differs from regression-based or structural equation modeling approaches, which primarily emphasize average linear relationships among variables.

The mean-split procedure was adopted to operationalize impulse-buying tendency within a theoretically driven segmentation framework. Three limitations of this approach are explicitly acknowledged. First, dichotomizing a continuous psychological construct introduces a degree of classification artificiality, such that observed group differences may partially reflect the imposed cut-point rather than naturally occurring consumer typologies (MacCallum et al., 2002). Second, the procedure may attenuate effect sizes and reduce statistical sensitivity to within-group variation (Iacobucci et al., 2015). Third, alternative analytical strategies—such as regression-based modeling, latent profile analysis, or cluster analysis—could reveal more nuanced consumer profiles, including multi-class structures that a binary classification cannot capture. Accordingly, the present findings should be interpreted as exploratory segmentation evidence rather than as precise estimates of continuous or multi-class effects, and future research employing these alternative approaches is encouraged to refine and extend the typological structure suggested here. Demographic characteristics of the final sample, including gender, impulse buying experience, primary retail channel, and main type of sport participation, are presented in Table 1.

### 5.2. Scale Validity and Reliability

The questionnaire consisted of 29 items designed to measure seven observed variables, excluding demographic characteristics: two single-factor constructs, impulse-buying tendency and self-justification, and five components of symbolic consumption tendency (self-development and reinforcement, conformity and belonging, self-formation, communication and exchange, and judgment and evaluation). Responses were evaluated on a 7-point Likert scale. Impulse-buying tendency, the independent variable, was measured using nine items from Verplanken and Herabadi (2001). Self-justification, the dependent variable, was assessed with four items adapted from Knox and Inkster (1968), and J. Kim (2022), while symbolic consumption tendency was measured using 16 items comprising five sub-factors adapted from Piacentini and Mailer (2004), J. Kim (2022), and Jeong and Jeon (2020).

Experts in sports marketing and consumer behavior verified the content validity of the questionnaire used in this study. Subsequently, confirmatory factor analysis (CFA) was conducted to establish convergent and discriminant validity. The hypothesized measurement model consisted of seven first-order latent constructs: impulse-buying tendency, self-justification, self-development and reinforcement, conformity and belonging, self-formation, communication and exchange, and judgment and evaluation. Each observed item was specified to load only onto its corresponding latent construct, and correlations among all latent variables were freely estimated. The CFA results showed that the fit indices of the measurement model satisfied the criteria for structural models proposed by Hu and Bentler (1999) (IFI > 0.90, CFI > 0.90, TLI > 0.90, RMSEA < 0.08, SRMR < 0.05), as shown in Table 2, confirming the acceptability of the measurement model.

To evaluate the possibility that the covariance among the observed indicators reflected a single measurement source, a CFA-based single-factor model was estimated by loading all items onto one latent construct and comparing it with the proposed multi-factor model. The results showed that the single-factor model fit the data substantially worse, indicating that a single common method source was unlikely to account for the relationships among the variables.

To examine the convergent validity of the constructs included in this study, construct reliability (CR) and average variance extracted (AVE) were computed. The CR values for the seven latent constructs ranged from 0.881 to 0.949, and the AVE values ranged from 0.675 to 0.856. Reliability estimates also indicated satisfactory internal consistency across all constructs, exceeding the recommended threshold of 0.70 (Nunnally, 1978), with McDonald’s ω ranging from 0.880 to 0.949 and Cronbach’s α ranging from 0.879 to 0.949 (see Table 2). Because all CR values were above 0.70 and all AVE values exceeded 0.50, the criteria suggested by Fornell and Larcker (1981) were satisfied, thereby supporting the convergent validity of the measurement model.

To assess discriminant validity, the AVE for each construct was compared with the squared correlations between constructs. Because the AVE values were greater than the corresponding squared inter-construct correlations in all cases, discriminant validity among the constructs was considered satisfactory (see Table 3).

### 5.3. Multivariate Analysis of Variance (MANOVA)

Prior to conducting the primary analyses, critical foundational assumptions were rigorously evaluated: (a) independence of observations, (b) randomness in sampling, and (c) equality of covariance matrices. The criteria for independence and random sampling were satisfied, as all randomly enlisted participants engaged with the questionnaire autonomously and without any overlap in participation. Additionally, the assumption concerning the homogeneity of the covariance matrices was empirically substantiated via Box’s M test.

MANOVA was conducted to examine differences in self-justification and symbolic consumption tendency between the high- and low-impulse-buying groups. First, Box’s M test indicated that the assumption of homogeneity of covariance matrices was not violated (Box’s M = 37.157, F = 1.720, *p* = 0.21), supporting the appropriateness of MANOVA for the present analysis. Furthermore, statistically significant differences were confirmed between the two groups (Wilks’ Lambda = 0.687, F = 17.231, *p* < 0.001, *ηp*^2^ = 0.313). The magnitude of effects was interpreted using conventional benchmarks for partial eta squared (*ηp*^2^), where 0.01, 0.06, and 0.14 indicate small, medium, and large effects, respectively (Richardson, 2011). The multivariate effect size (*ηp*^2^ = 0.313) substantially exceeded the conventional threshold for a large effect, indicating that approximately 31% of the multivariate variance in the composite dependent variable set was attributable to impulse-buying tendency group membership. This magnitude suggests that impulse-buying tendency operates not as a marginal correlate but as a substantively meaningful basis of dispositional segmentation within South Korean Gen Z sports consumers.

Because the multivariate effect was statistically significant, follow-up univariate ANOVAs identified the specific dependent variables driving this difference. To curb the inflation of familywise Type I error across the six distinct dependent measures, a rigorous Bonferroni-adjusted alpha criterion of 0.008 (0.05/6) was instituted (Dunn, 1961). Univariate tests revealed significant group differences in self-justification, self-development and reinforcement, conformity and belonging, and communication and exchange. Self-formation, which was initially significant, did not remain significant after Bonferroni correction, and judgment and evaluation was not statistically significant (see Table 4). A comparison of effect sizes across dependent variables reveals a clear ordering of magnitude. Self-justification (*ηp*^2^ = 0.204) and conformity and belonging (*ηp*^2^ = 0.199) showed the largest group differences, each accounting for approximately one-fifth of the variance in their respective constructs—a substantial magnitude indicating that high and low impulse-buying consumers represent qualitatively distinct populations on these dimensions. Self-development and reinforcement (*ηp*^2^ = 0.091) showed a moderate effect, suggesting a meaningful but less pronounced separation, while communication and exchange (*ηp*^2^ = 0.044) showed a small-to-moderate effect that, although statistically significant, indicates only partial group differentiation. The non-significant effects for self-formation and judgment and evaluation (*ηp*^2^ = 0.028 and 0.005, respectively) represent negligible group separation, with less than 3% of variance attributable to impulse-buying tendency group membership. Furthermore, as delineated in Table 5, the high impulse-buying cohort consistently exhibited elevated mean values relative to their low-impulse counterparts across the entire spectrum of dependent constructs.

## 6. Discussion

The present findings suggest that impulse-buying tendency functions not merely as a linear predictor of consumer outcomes, but as a meaningful dispositional criterion for distinguishing qualitatively different psychological and symbolic consumption profiles within sports consumer markets. This perspective extends current understanding beyond a purely predictive behavioral framework toward a psychologically differentiated segmentation approach in sports consumption research. This perspective challenges the dominant assumption that impulse buying linearly predicts consumer outcomes. Instead, the present findings suggest that impulse-buying tendency may correspond to qualitatively different psychological meaning systems related to post-purchase cognition and symbolic consumption. In this respect, the study extends current understanding of impulse buying from a primarily predictive behavioral construct toward a psychologically differentiated consumer segmentation framework within sports consumption contexts.

Therefore, this study examined whether levels of impulse-buying tendency differentiate self-justification and symbolic consumption tendency among South Korean Gen Z sports consumers. The findings revealed that the high impulse-buying tendency group reported consistently higher mean scores across all dependent variables, with statistically significant differences confirmed for self-justification, self-development and reinforcement, conformity and belonging, and communication and exchange. These results indicate that, even within the same generational cohort, impulse-buying tendency serves as a meaningful basis for distinguishing post-purchase cognitive responses and identity-related consumption patterns. In particular, the findings suggest that consumers with higher impulse-buying tendency exhibited stronger tendencies to rationalize their purchase decisions after consumption to use sports product consumption as a means of self-expression and social positioning. Accordingly, the present study extends the understanding of impulse-buying tendency beyond a purely behavioral disposition by demonstrating its relevance to psychologically and symbolically meaningful differences in sports consumption.

### 6.1. Theoretical Implications

This study offers several important theoretical implications for consumer behavior and sports consumption research. First, the findings extend the literature on impulse-buying tendency by showing that this construct should not be understood solely as a dispositional tendency toward spontaneous purchasing, but also as a meaningful segmentation criterion that differentiates post-purchase cognition and symbolic consumption patterns among South Korean adult Gen Z sports consumers. Prior studies have typically examined impulse buying within causal or predictive frameworks, focusing on its antecedents or consequences as a continuous behavioral trait (Amos et al., 2014; Beatty & Ferrell, 1998; Iyer et al., 2020). In contrast, the present study adopted a group-comparison approach and demonstrated that consumers with higher and lower impulse-buying tendency exhibit systematically different psychological and symbolic consumption profiles. In this sense, the findings suggest that impulse-buying tendency functions not merely as a purchasing disposition, but as a meaningful basis for identifying heterogeneity within a single generational cohort. Unlike regression-based or structural modeling approaches that primarily estimate linear predictive relationships among variables, the present study adopts a segmentation-based perspective to examine whether consumers with different levels of impulse-buying tendency exhibit distinct psychological and symbolic consumption profiles. This approach allows the identification of dispositional heterogeneity within Generation Z sports consumers rather than focusing solely on average predictive effects. Although this segmentation-based approach offers interpretive advantages for identifying dispositional heterogeneity, it should be complemented by continuous or multi-class analytical strategies in future research to validate and extend the consumer typologies suggested here. Accordingly, the present study suggests that sports consumer behavior may be more effectively interpreted through differentiated psychological profiles rather than through uniform predictive relationships alone. This perspective may encourage future sports consumer research to incorporate segmentation-oriented interpretations alongside traditional regression-based approaches.

Second, from the perspective of CDT, the significant difference in self-justification between the high and low impulse-buying tendency groups indicates that impulsive consumption is closely associated with post-purchase cognitive regulation. Consumers with higher impulse-buying tendency were more likely to reinterpret and defend their purchase decisions, suggesting that the psychological meaning associated with impulsive purchasing may extend beyond the point of acquisition. Rather, it was associated with stronger patterns of post-purchase cognitive regulation aimed at restoring internal consistency and reducing psychological tension. This pattern may reflect a stronger tendency among high impulse-buying consumers to restore cognitive consistency following emotionally driven purchasing decisions. Crucially, the present findings extend this theoretical proposition by demonstrating that the intensity of self-justification differs systematically between consumer groups. This suggests that impulse-buying tendency does not merely trigger dissonance-reduction in a binary fashion; rather, it conditions the magnitude of post-purchase cognitive regulation, with higher dispositional impulsivity corresponding to stronger rationalization tendencies. The present findings are also consistent with prior consumer behavior studies reporting that consumers with stronger impulsive purchasing tendencies tend to engage more actively in post-purchase rationalization processes to reduce psychological discomfort and maintain cognitive consistency (Dhandra, 2020; Rook & Fisher, 1995). More specifically, the results imply that the intensity of post-purchase rationalization differed between consumers classified into high and low impulse-buying tendency groups toward impulsive purchasing. Thus, the present findings indicate that impulse-buying tendency may operate as a conditioning layer that corresponds to differences in post-decision dissonance-related responses and cognitively mediated rationalization processes. Notably, the effect size for self-justification (*ηp*^2^ = 0.204) was the largest among all dependent variables and substantially exceeded the conventional large-effect threshold (0.14). This magnitude suggests that the cognitive regulation pathway proposed by CDT is particularly strongly activated among high impulse-buying consumers, reinforcing the theoretical claim that dispositional impulsivity systematically intensifies post-purchase cognitive consistency restoration. By comparison, prior consumer dissonance research has typically reported small to moderate effects (*ηp*^2^ < 0.10) for dispositional moderators (Akbar et al., 2020; Saranya & Joji Alex, 2024), positioning the present finding as a relatively pronounced manifestation of the dispositional–dissonance link.

Third, the findings refine the theoretical understanding of symbolic consumption in sports markets. Significant group differences were found in self-development and reinforcement, conformity and belonging, and communication and exchange, suggesting that consumers with higher impulse-buying tendency are more likely to use sports product consumption as a means of self-enhancement, group affiliation, and image communication. As established theoretically, sports products operate as visible symbolic markers; The effect-size pattern further refines this interpretation. Among the three significantly differentiated symbolic consumption dimensions, conformity and belonging exhibited the largest group difference (*ηp*^2^ = 0.199, large), followed by self-development and reinforcement (*ηp*^2^ = 0.091, medium) and communication and exchange (*ηp*^2^ = 0.044, small-to-medium). This ordering suggests that group-affiliative symbolic motives are most strongly activated by high impulse-buying disposition, followed by self-enhancement and expressive communication motives. In terms of practical interpretation, the large effect for conformity and belonging implies that high impulse-buying consumers are not merely somewhat more but substantially more oriented toward group-belonging symbolic consumption than low impulse-buying consumers—a difference in practical magnitude rather than statistical artifact. In particular, the results align with SSCT by suggesting that sports products may function as symbolic resources through which consumers reinforce a desired self-image, signal belonging to meaningful groups, and communicate socially valued aspects of the self (Rincón et al., 2023; Wicklund & Gollwitzer, 1982; Yu et al., 2020).

At the same time, the dimension-specific pattern of significant and non-significant differences warrants deeper theoretical consideration. Although the high impulse-buying tendency group showed higher mean scores across all dependent variables, self-formation did not survive the Bonferroni correction, and judgment and evaluation were not statistically significant. This selective pattern is not theoretically arbitrary; rather, it can be interpreted through dual-process accounts of consumer cognition (Strack & Deutsch, 2004) and structural models of the self-concept (Markus & Nurius, 1986; Markus & Wurf, 1987; Sirgy, 1982).

According to the Reflective-Impulsive Model (Strack & Deutsch, 2004), consumer behavior is jointly governed by an impulsive system, which operates through automatic, affect-laden, and associatively activated processes, and a reflective system, which operates through deliberative, propositional, and evaluative processes. Impulse-buying tendency, by definition, indexes individual differences in the relative dominance of the impulsive system over the reflective system (Verplanken & Sato, 2011; Vohs & Faber, 2007). It follows that symbolic consumption dimensions psychologically aligned with the impulsive system—those activated by immediate emotional cues, peer comparison, and socially expressive impulses—should be most strongly differentiated by impulse-buying tendency. Self-development and reinforcement, conformity and belonging, and communication and exchange map closely onto this profile: each involves affectively charged, situationally cued, and outwardly expressive consumption motives that the impulsive system can readily mobilize without extensive deliberation.

In contrast, self-formation and judgment and evaluation engage qualitatively different psychological processes. Self-formation reflects the construction of relatively stable identity structures—what self-concept research describes as the core self or the structural component of the working self-concept (Markus & Nurius, 1986; Markus & Wurf, 1987). Because the core self is integrated over time and resistant to momentary situational influences (Sirgy, 1982), it is unlikely to be substantially differentiated by a dispositional tendency oriented toward immediate, situationally triggered purchasing responses. Judgment and evaluation, in turn, involves inferential processing of others’ status, social background, and consumption signals—a task that recruits the reflective rather than the impulsive system, as it requires categorical reasoning and propositional evaluation (Strack & Deutsch, 2004). Such reflectively governed processes are, by construction, less responsive to dispositional impulsivity.

Consistent with the Identity-Based Motivation framework (Oyserman, 2009), this pattern can be further understood as a function of identity accessibility: socially expressive and group-affiliative identities (associated with SR, CB, and CE) are readily activated by situational cues common in sports consumption contexts—peer visibility, brand environments, and digital social interaction—whereas deeper identity-construction processes (SF) and other-directed social inferences (JE) require more sustained reflective engagement that impulsive disposition does not preferentially activate. Furthermore, the self-regulatory resource depletion that characterizes high impulse-buying consumers (Vohs & Faber, 2007) tends to facilitate processes already aligned with the impulsive system while constraining those requiring reflective elaboration—a mechanism consistent with the asymmetric pattern observed here.

Taken together, these theoretical considerations suggest that the relationship between impulse-buying tendency and symbolic consumption is not uniformly distributed across dimensions but is systematically channeled through impulsive-system-compatible motives. This refines the conventional treatment of symbolic consumption as an internally integrated construct (McCracken, 1986; Piacentini & Mailer, 2004) and supports a more differentiated, process-based view in which dispositional impulsivity selectively amplifies symbolic motives that the impulsive system can readily mobilize. The findings therefore extend prior symbolic consumption research by demonstrating that dimensional asymmetry is theoretically predictable rather than empirically incidental.

Fourth, the present study contributes to the growing literature on intra-generational heterogeneity within Gen Z. Gen Z is often treated as a relatively homogeneous consumer cohort because of its shared exposure to digital environments, social media, and rapid consumption culture (Theocharis, 2025). However, the present findings suggest that even within this same cohort, meaningful differences emerge according to impulse-buying tendency. This point is theoretically important because it challenges the tendency to treat Gen Z as a monolithic market segment. Instead, the results indicate that variation in consumption disposition within a single generation may lead to distinct post-purchase cognitive responses and symbolic consumption orientations. Therefore, the present study supports a more differentiated understanding of Gen Z sports consumers by emphasizing dispositional segmentation rather than broad generational generalization. It should be noted, however, that because the sample was drawn from physically active, university-linked, sport-engaged populations, the symbolic consumption patterns observed here may be more pronounced than those characterizing broader Gen Z populations. The dispositional heterogeneity documented in this study should therefore be understood as variation within an already identity-engaged sports consumer subgroup, rather than as a description of Gen Z sports consumers at large.

Finally, the present study contributes to sports consumer research by showing that sports product consumption should be interpreted not only as a functional or transactional activity, but also as a psychologically and symbolically layered process. By integrating impulse-buying tendency, self-justification, and symbolic consumption tendency within a single comparative framework, this study demonstrates that sports consumption is shaped by both post-purchase cognitive adjustment and identity-related symbolic motives. In this respect, the findings extend existing work on sports consumer behavior by linking impulsive acquisition to both dissonance reduction and symbolic meaning construction. More broadly, they suggest that future behavioral research on sports consumption should pay closer attention to how impulsive dispositions interact with post-purchase cognition and symbolic motives, rather than treating these domains as analytically separate.

### 6.2. Practical Implications

Based on the findings of the present study, several practical implications can be drawn for sports product marketing and consumer engagement. The results suggest that impulse-buying tendency serves as a meaningful segmentation criterion in sports markets, with the magnitude of group differences providing additional guidance for resource allocation. In particular, the large effect sizes for self-justification and conformity and belonging (*ηp*^2^ = 0.20) indicate that marketing investments aimed at post-purchase reassurance communication and group-affiliative symbolic positioning are likely to yield the most pronounced differential impact between high and low impulse-buying consumer segments. Conversely, the smaller effects for self-development and reinforcement (*ηp*^2^ = 0.091) and communication and exchange (*ηp*^2^ = 0.044) suggest that these symbolic dimensions, while meaningfully differentiated, warrant proportionally more modest segmentation-based emphasis. In particular, consumers with higher impulse-buying tendency showed stronger self-justification and higher levels of several dimensions of symbolic consumption tendency, indicating that effective marketing strategies should address not only immediate purchase stimulation but also post-purchase reassurance and identity-related symbolic value.

First, it may be beneficial for sports brands and retailers to design differentiated communication strategies according to levels of impulse-buying tendency. Because the high impulse-buying tendency group showed significantly higher levels of self-justification, marketers should not focus solely on stimulating the initial purchase, but also on reinforcing consumers’ confidence after purchase. For example, post-purchase communication may emphasize product quality, performance benefits, appropriateness of choice, or compatibility with the consumer’s lifestyle and sporting goals. Such strategies may help consumers cognitively validate their purchases and reduce possible post-purchase tension (Beikverdi et al., 2024). In this sense, effective marketing does not end at the point of sale, but extends into the post-purchase stage through reassurance-oriented communication.

Second, the findings suggest that sports product marketing should emphasize symbolic and identity-related values rather than relying exclusively on functional attributes. Significant group differences were found in self-development and reinforcement, conformity and belonging, and communication and exchange, indicating that consumers with higher impulse-buying tendency are more likely to use sports product consumption as a means of self-enhancement, social affiliation, and expressive communication. Accordingly, sports brands may benefit from framing their products not only as performance tools, but also as resources for confidence building, community connection, and identity expression. Messages that highlight improvement of the self, belonging to a desired group, and the communication of one’s image may be particularly effective for consumers with stronger impulsive tendencies (Chauhan et al., 2021).

Third, a more segmented retail and promotional strategy may be required across digital and offline sports consumption environments. Consumers with high impulse-buying tendency may be more responsive to emotionally vivid imagery, socially visible brand cues, limited-time promotions, influencer-based endorsements, and community-linked product meanings (Redine et al., 2023). By contrast, consumers with lower impulse-buying tendency may respond more favorably to information-rich and deliberation-oriented appeals centered on product functionality, durability, and value (Escobar-Farfán et al., 2025). This suggests that sports brands should move beyond uniform promotional tactics and instead tailor message framing, content presentation, and channel strategy according to dispositional consumer differences.

Fourth, the present findings imply that sports product environments should be designed to support socially meaningful and psychologically reassuring consumption experiences. Because communication and exchange emerged as a significant dimension, sports brands may strengthen consumer engagement by creating opportunities for consumers to share, display, and socially connect through their purchases (Zollo et al., 2020). For example, digital platforms linked to sports brands may encourage consumers to communicate their identities through user-generated content, peer interaction, and brand community participation (Jacobson et al., 2020). Similarly, offline retail environments may be structured to enhance visibility, affiliation, and self-expression through experiential displays, community campaigns, and identity-relevant storytelling (Alexander & Cano, 2020). Such approaches may be particularly effective for South Korean Gen Z sports consumers, who are highly attuned to peer visibility, symbolic meaning, and relational signaling.

At the same time, the practical implications of the present study should be interpreted with appropriate caution. The non-significant findings for self-formation and judgment and evaluation suggest that not all aspects of symbolic consumption tendency are equally influenced by impulse-buying tendency. Therefore, practitioners should avoid assuming that all symbolic motives will be activated uniformly among high impulse-buying consumers. Instead, the findings support a more selective application of symbolic marketing strategies, particularly those related to self-reinforcement, group belonging, and interpersonal expression.

Taken together, the present study suggests that effective sports marketing should connect immediate purchase stimulation with post-purchase cognitive reinforcement and identity-relevant symbolic communication. In this respect, impulse-buying tendency may serve as a useful practical basis for consumer segmentation, allowing brands to distinguish not only who is more likely to buy impulsively, but also how those consumers are likely to rationalize, interpret, and symbolically use their purchases after acquisition. These practical implications are most directly applicable to marketing contexts targeting physically active, identity-engaged Gen Z sports consumers—such as performance-oriented brand campaigns, fitness community platforms, and participation-linked retail environments. Their relevance to broader Gen Z populations, including casual or non-participatory sports consumers, requires further empirical validation.

## 7. Conclusions

The present study examined whether differences in self-justification and symbolic consumption tendency emerge according to impulse-buying tendency among adult South Korean Generation Z sports consumers. The findings revealed that consumers classified into the high impulse-buying tendency group exhibited significantly higher levels of self-justification, self-development and reinforcement, conformity and belonging, and communication and exchange compared with the low impulse-buying tendency group. By contrast, self-formation and judgment and evaluation did not show statistically significant group differences after Bonferroni correction. These findings suggest that impulse-buying tendency may be selectively associated with certain psychologically and socially expressive dimensions of symbolic consumption rather than with symbolic consumption tendency as a uniformly integrated construct.

The present study demonstrated that impulse-buying tendency constitutes a meaningful dispositional segmentation criterion among adult South Korean Generation Z sports consumers. Specifically, high impulse-buying tendency was associated with heightened self-justification and stronger engagement in self-development and reinforcement, conformity and belonging, and communication and exchange dimensions of symbolic consumption. These findings indicate that post-purchase cognitive regulation and symbolic identity practices are not uniformly distributed within a generational cohort, but vary systematically according to impulsive disposition.

Theoretically, these results refine both CDT and SSCT by specifying the conditions under which their central propositions operate most strongly—namely, among consumers whose purchasing behavior is dispositionally characterized by limited pre-purchase deliberation. The selective, dimension-specific pattern of symbolic consumption differences further challenges the assumption that symbolic motives operate as a homogeneous construct, suggesting instead that only socially expressive and self-reinforcing dimensions are closely linked to impulsive disposition in sports consumption contexts.

From a practical standpoint, impulse-buying tendency may serve as an actionable segmentation basis, enabling sports marketers to design differentiated communication strategies that address both post-purchase reassurance and identity-affirming symbolic value. Future research should examine these relationships longitudinally, across diverse cultural contexts, and using continuous variable approaches to overcome the limitations of the present cross-sectional, mean-split design.

From a practical perspective, the findings imply that sports product marketing strategies targeting adult Generation Z consumers may benefit from more differentiated approaches that account for post-purchase cognition, symbolic consumption orientation, and emotionally responsive consumption tendencies. In particular, strategies emphasizing identity expression, social belonging, and psychologically reinforcing consumption experiences may be more effective for consumers with higher impulse-buying tendency.

## 8. Limitations and Future Research

This study has several limitations. First, generalizability is constrained by both demographic and recruitment-channel characteristics of the sample. Because recruitment was conducted through universities, sports centers, and golf facilities, the sample overrepresents three theoretically consequential subgroups: (a) consumers with pre-existing sport interest, (b) university-affiliated young adults, and (c) consumers who have already committed to a sport as an identity-relevant practice. This recruitment design may have inflated the observed levels of symbolic consumption tendency relative to what would be observed in a broadly representative Gen Z sample that included sedentary consumers, non-students, and individuals with no prior sport involvement. Consequently, the group-difference patterns identified here should not be assumed to characterize the full Gen Z population; rather, they characterize identity-engaged, physically active Gen Z sports consumers within the South Korean context. In addition, because the study included only legal adults aged 20 years or older, the findings primarily reflect older Gen Z consumers and may not generalize to adolescents or financially dependent minors, whose purchasing autonomy, disposable income, and consumption accessibility differ substantially. Future research employing probability-based sampling, online panels that include non-participatory sports consumers, and cross-national designs is needed to clarify the extent to which the present patterns hold beyond identity-engaged, university-linked, physically active subgroups. Second, because the study relied on self-reported survey data, the results reflect perceived rather than actual consumption behavior. Third, although sports product consumption was examined broadly, the meaning and function of consumption may differ across product categories, such as sports apparel, team merchandise, and performance equipment. Fourth, although the rationale and limitations of the mean-split procedure are discussed in Section 5.1, future research should examine impulse-buying tendency as a continuous construct using regression-based modeling, or alternatively employ latent profile or cluster analyses to identify multi-class consumer typologies that a binary classification cannot capture. Finally, because the study employed a cross-sectional design, causal relationships cannot be inferred. Future research should therefore examine more diverse populations and product categories, incorporate behavioral or experimental data, and analyze impulse-buying tendency as a continuous construct using longitudinal or model-based approaches.

## Figures and Tables

**Table 1:** Descriptive statistics of survey respondents.

		Group 1	Group 2
Impulse Buying Level		High	Low
Gender	Male	60 (56.6%)	80 (62.5%)
	Female	46 (43.4%)	48 (37.5%)
Impulse buying experience	Yes	56 (52.8%)	36 (28.1%)
	No	47 (44.3%)	92 (71.9%)
Primary retail channel	Offline stores	39 (36.8%)	54 (42.2%)
	Online stores	56 (52.8%)	61 (47.7%)
	Official brand websites	6 (5.7%)	10 (7.8%)
	Secondhand trading platforms	5 (4.7%)	3 (2.3%)
Main type of sport participation	Golf	30 (28.3%)	37 (28.9%)
	Fitness	32 (30.2%)	19 (14.8%)
	Running	18 (17.0%)	20 (15.6%)
	Yoga	8 (7.5%)	12 (9.4%)
	Ball sports	3 (2.8%)	19 (14.8%)
	Others	15 (14.2%)	21 (16.4%)
Total		106 (100%)	128 (100%)

Note. % = Amount in each hundred in each group.

**Table 2:** Result of the confirmatory factor analysis.

Factors	Items				*β*	S.E.		*t*	CR	AVE	ω	α
Impulse buying tendency	I often buy sports products spontaneously.				0.775	-		-	0.949	0.675	0.949	0.949
	“Just do it” describes the way I buy sports products.				0.736	0.079		12.096				
	I often buy sports products without thinking.				0.861	0.077		14.742				
	“I see it, I buy it” describes me when purchasing sports products.				0.803	0.080		13.475				
	“Buy now, think about it later” describes me when purchasing sports products.				0.847	0.081		14.419				
	Sometimes I feel like buying sports products on the spur of the moment.				0.796	0.077		13.312				
	I buy sports products according to how I feel at the moment.				0.884	0.077		15.266				
	I tend not to carefully plan most of my sports product purchases.				0.839	0.076		14.244				
	Sometimes I am a bit reckless about the sports products I buy.				0.842	0.082		14.313				
Self-justification	I tried to focus more on the strengths than the weaknesses of the sports product I purchased.				0.878	-		-	0.907	0.710	0.908	0.907
	I thought that other sports products would probably have been similar.				0.865	0.051		17.237				
	I believed that the sports product I chose was the best option at that time.				0.807	0.060		15.367				
	I tried to find positive aspects of the sports product I purchased.				0.819	0.055		15.771				
Self-development and reinforcement	Through consuming sports products, I can develop different aspects of myself.				0.816	-		-	0.923	0.708	0.923	0.923
	Consuming sports products is an important means of improving myself.				0.878	0.068		16.259				
	Through consuming sports products, I create a confident image of myself.				0.912	0.065		17.216				
	I purchase sports products that make me feel like a valuable person.				0.809	0.069		14.417				
	I consume sports products in order to create a better version of myself.				0.784	0.066		13.770				
Conformity and belonging	I try to consume sports products similar to those used by the group I want to belong to.				0.843	-		-	0.892	0.735	0.880	0.879
	I tend to prefer trendy sports products.				0.861	0.067		15.656				
	I tend to be sensitive to the sports products or brands owned by people around me.				0.867	0.077		14.798				
Self-formation	My consumption of sports products reflects what kind of person I am.				0.880	-		-	0.881	0.711	0.906	0.905
	Through consuming sports products, I can distinguish myself from others.				0.841	0.065		16.869				
	Consuming sports products is a means of shaping my image.				0.807	0.063		16.041				
Communication and exchange	I try to communicate my image through consuming sports products.				0.829	-		-	0.906	0.763	0.893	0.890
	I try to reveal my strengths through consuming sports products.				0.918	0.070		15.638				
	I express my values through consuming sports products.				0.872	0.066		15.784				
Judgment and evaluation	A person’s consumption of sports products serves as a basis for inferring that person’s status.				0.928	-		-	0.922	0.856	0.922	0.922
	A person’s sports products make it possible to infer that person’s social background.				0.922	0.064		15.603				
Model fit	χ^2^	*df*	*p*	IFI	TLI		CFI		SRMR		RMSEA	
	2.285	356	<0.001	0.918	0.906		0.918		0.044		0.074	

**Table 3:** Descriptive statistics and correlations between key variables.

Classification	1	2	3	4	5	6	7
Impulse buying tendency	**0** **.822**						
Self-justification	0.380 **	**0** **.843**					
Self-development and reinforcement	0.361 **	0.257 **	**0** **.841**				
Conformity and belonging	0.472 **	0.356 **	0.590 **	**0** **.857**			
Self-formation	0.197 **	0.162 **	0.493 **	0.293 **	**0** **.843**		
Communication and exchange	0.264 **	0.208 **	0.501 **	0.378 **	0.319 **	**0** **.873**	
Judgment and evaluation	0.130 *	0.230 **	0.338 **	0.260 **	0.301 **	0.538 **	**0** **.925**
Mean	3.45	4.36	3.44	3.81	3.90	4.18	4.82
Standard Deviation	1.53	1.21	1.47	1.37	1.48	1.43	1.30
Skewness	0.349	−0.136	0.038	−0.137	−0.153	−0.074	−0.652
Kurtosis	−0.717	−0.478	−0.770	−0.333	−0.477	−0.424	0.733

Note. ** *p* < 0.01, * *p* < 0.05, Bold = $\sqrt{AVE}$.

**Table 4:** Results of multivariate analysis of variance.

Variables	Sub-Factors	*df*	F	*p*	*ηp* ^2^
Self-Justification		1	70.227	<0.001	0.204
Symbolic consumption tendency	Self-development and reinforcement	1	45.716	<0.001	0.091
	Conformity and belonging	1	87.667	<0.001	0.199
	Self-formation	1	14.376	0.010	0.028
	Communication and exchange	1	20.877	0.001	0.044
	Judgment and evaluation	1	2.114	0.264	0.005

Note. Follow-up univariate tests were interpreted using a Bonferroni-adjusted alpha level of 0.008 (0.05/6).

**Table 5:** Mean scores by group.

	a	b	c	d	e	f
G1	4.96	3.92	4.49	4.17	4.51	4.93
G2	3.86	3.03	3.26	3.67	3.91	4.74

Note. G1 = High impulse buying tendency, G2 = Low impulse buying tendency. a = Self-Justification, b = Self-development and reinforcement, c = Conformity and belonging, d = Self-formation, e = Communication and exchange, f = Judgment and evaluation.

## Data Availability

The data presented in this study are available on request from the corresponding author. The data are not publicly available due to ethical constraints and the need to safeguard participants’ privacy.

## Abbreviations

The following abbreviations are used in this manuscript:

AVE	Average Variance Extracted
CB	Conformity and belonging
CDT	Cognitive Dissonance Theory
CE	Communication and exchange
CFA	Confirmatory Factor Analysis
CFI	Comparative Fit Index
CR	Construct Reliability
Gen Z	Generation Z
IBT	Impulse buying tendency
IFI	Incremental Fit Index
IRB	Institutional Review Board
JE	Judgment and evaluation
MANOVA	Multivariate Analysis of Variance
RMSEA	Root Mean Square Error of Approximation
SD	Standard Deviation
SE	Standard Error
SF	Self-formation
SJ	Self-justification
SR	Self-development and reinforcement
SRMR	Standardized Root Mean Square Residual
SSCT	Symbolic Self-Completion Theory
TLI	Tucker–Lewis Index
